# The Infant Feeding Activity and Nutrition Trial (INFANT) an early intervention to prevent childhood obesity: Cluster-randomised controlled trial

**DOI:** 10.1186/1471-2458-8-103

**Published:** 2008-03-31

**Authors:** Karen Campbell, Kylie Hesketh, David Crawford, Jo Salmon, Kylie Ball, Zoë McCallum

**Affiliations:** 1 Centre for Physical Activity and Nutrition Research, Faculty of Health, Medicine, Nursing and Behavioral Sciences, Deakin University, Victoria, Australia; 2 Department of Pediatrics, University of Melbourne, Victoria, Australia

## Abstract

**Background:**

Multiple factors combine to support a compelling case for interventions that target the development of obesity-promoting behaviours (poor diet, low physical activity and high sedentary behaviour) from their inception. These factors include the rapidly increasing prevalence of fatness throughout childhood, the instigation of obesity-promoting behaviours in infancy, and the tracking of these behaviours from childhood through to adolescence and adulthood. The Infant Feeding Activity and Nutrition Trial (INFANT) aims to determine the effectiveness of an early childhood obesity prevention intervention delivered to first-time parents. The intervention, conducted with parents over the infant's first 18 months of life, will use existing social networks (first-time parent's groups) and an anticipatory guidance framework focusing on parenting skills which support the development of positive diet and physical activity behaviours, and reduced sedentary behaviours in infancy.

**Methods/Design:**

This cluster-randomised controlled trial, with first-time parent groups as the unit of randomisation, will be conducted with a sample of 600 first-time parents and their newborn children who attend the first-time parents' group at Maternal and Child Health Centres. Using a two-stage sampling process, local government areas in Victoria, Australia will be randomly selected at the first stage. At the second stage, a proportional sample of first-time parent groups within selected local government areas will be randomly selected and invited to participate. Informed consent will be obtained and groups will then be randomly allocated to the intervention or control group.

**Discussion:**

The early years hold promise as a time in which obesity prevention may be most effective. To our knowledge this will be the first randomised trial internationally to demonstrate whether an early health promotion program delivered to first-time parents in their existing social groups promotes healthy eating, physical activity and reduced sedentary behaviours. If proven to be effective, INFANT may protect children from the development of obesity and its associated social and economic costs.

**Trial registration:**

Current Controlled Trials ISRCTN81847050

## Background

Preventing the development of obesity in children is an international health priority [1]. Current estimates suggest that the prevalence of overweight and obesity in all age groups is rapidly increasing worldwide [2]. In Australia approximately 25% of children are overweight or obese and that estimate is growing with data highlighting that these increases begin in early childhood [3]. An Australian sample of 114,669 pre-schoolers showed the prevalence of overweight and obesity increased from 16.3 to 27.2 per cent in girls and from 13.4 to 21.4 per cent in boys between 1995 and 2002 [4].

Overweight and obesity are recognised to have numerous negative impacts on children's health and wellness during childhood and through to adult life [5,6]. Further, research has shown that adiposity within the childhood period is a stable trait [7] and that parents are often poor at identifying fatness in their children [8]. In addition, obese children tend to become obese adults and treatment is difficult and costly [2]. Cochrane reviews have established that opportunities for prevention are poorly understood [9]. Overall there is an urgent need for research on the capacity to influence the development of children's obesity- promoting behaviours in early life.

Overweight in early childhood is determined in part by eating, physical activity and sedentary behaviours learnt at home in the first five years of life. The intervention outlined is informed by the understanding that (a) obesity-promoting behaviours are established early in life, (b) parents play a primary role in shaping these behaviours in infancy, (c) intervening before these behaviours (and parents responses to them) are established is likely to be effective, and (d) that the social milieu provided by parent groups is likely to facilitate and support the uptake of health promotion messages.

### Obesity-promoting behaviours are established early in life

Recent evidence highlights that obesity-promoting dietary habits, such as high consumption of energy-dense foods and fluids, previously documented in children [10,11] are also evident in infants and toddlers [12]. Australian data using 3-day weighed food records (n = 538), shows 90% of 18-month old children consumed energy-dense snack foods on the recall days and 70% consumed sweetened non-milk drinks (e.g. soft drinks) [13]. Overall, foods considered as "extra" or "non-core" provided 27% of the total energy intake in that sample. In addition, the nutritional quality of diet is known to continue to decline throughout childhood and adolescence [14,15].

These early dietary patterns couple with evidence of high levels of sedentary behaviours in early childhood. For example, 17% of 0–11 month and 48% of 12–23 month old children in the US watch more than the recommended two hours of television per day, and this proportion increases throughout childhood [16]. Further evidence suggests that viewing television for more than two hours per day is positively associated with obesity-promoting dietary behaviours and low levels of physical activity in young children [17,18]. Little physical activity trend data during infancy and early childhood is available, however it appears that physical activity levels fall throughout this period [19,20].

In addition to population trend data, there is evidence of tracking of children's dietary [21], sedentary and physical inactivity behaviours [22] from childhood to adolescence and adulthood, which appear to impact on adult health [23]. Thus, the obesity-promoting behaviours learned and supported during these early years may establish lifestyle behaviours that will track throughout the lifespan. Given this, it is reasonable to posit that early childhood provides a unique and circumscribed opportunity within which to establish lifestyle behaviours that will promote health and minimize the risk of the development of obesity.

### The role of parents in the promotion of children's behaviours

One of the most powerful predictors of weight management in overweight children is parental involvement, yet there remains an urgent need to examine opportunities to prevent childhood overweight and obesity via parental involvement in the early years [24-26]. Children's eating, physical activity and sedentary behaviours are learnt and sustained in the home and there is evidence that this environment impacts on children's weight [27,28]. Parents have the capacity via their nutrition knowledge, parenting style, modelling and the food environment to impact on children's emerging food choices [27]. Evidence has shown that in a young population (2–6 year olds, n = 564) the strongest predictor of children's fruit and vegetable consumption was parent consumption [29]. In addition, child rejection of fruits and vegetables (negative association) was modifiable with repeated exposure to rejected foods.

In terms of parents' involvement in physical activity with their children, one study reports that parents spend 13% of their child's play time in active play with their infant and that the remainder of time is spent in object play [30]. The use of parks and outdoor spaces is reported by less than half of parents with 5–12 year olds [20]. Children whose parents are active with them are reported to have higher levels of physical activity [31,32]. However, family rules are reported to be inversely associated with children's physical activity [26]. Similarly, rules prohibiting television viewing during mealtimes are reported to be inversely associated with children's television viewing time, and frequency of parent's watching television with their child has been found to be positively associated with children's television viewing [33].

Despite these trends, first-time parents may be particularly receptive to knowledge and skill development around parenting and the promotion of healthy family eating and physical activity behaviours. First-time parents regularly seek advice during their child's first year of life. In Victoria Australia, families make approximately 35 visits to health care providers for their infants during this first year [34]. Importantly, most visits to health service providers are not related to child illness, but rather reflect parental need for support and information during this period of rapid transition. In addition, parents indicate high levels of concern regarding children's appetite, eating patterns and growth, and regularly express the need for more comprehensive guidance in these areas [35]. It is likely that messages delivered to first-time parents may be preferentially received if delivered at times in their child's development when they are actively seeking strategies to manage emerging behaviours, an approach known as anticipatory guidance.

### Utilisation of anticipatory guidance

Anticipatory guidance is heralded as a promising approach by which health practitioners might support parents to promote healthy weight in their children by being proactive, informing parents about what to expect and how to manage behaviour, as opposed to supporting parents to manage events after they occur [36-38]. Anticipatory guidance has been shown to be effective across a range of domains, including parent-infant interactions, sleep patterns, injury prevention and reading at home [39].

Despite the promise of anticipatory guidance as an educative approach, just one study utilising this approach in the area of childhood eating has been published [40]. That study involved guidance of parents of new-borns regarding delaying introduction of solids. Compared to controls, the intervention resulted in positive differences in the types of foods introduced and increased confidence in health professionals as primary providers of information.

A randomised-controlled trial targeting overweight indigenous mothers of 1–3 year olds focused on parenting skills to promote improved child eating and physical activity patterns [41]. That study found intervention group infants had decreased relative weight, total energy intake, and improved parent-child interactions around food over 16 weeks. This high quality US study highlights that parents are willing and capable of making positive changes to improve their child's health and body weight.

### INFANT: preventing childhood obesity and promoting healthy life-style choices

The INFANT project will employ an anticipatory guidance approach to support first-time parents attending a new parents' group to be skilled in their approaches to their infant's emerging dietary, physical activity and sedentary behaviours. The intervention will be delivered by an experienced dietitian during infants' first 18 months of life at first-time parents groups held within Maternal and Child Health (MCH) centers. Evidence supports the proposition that education regarding lifestyle behaviours is feasible within existing MCH infrastructures and that it is likely to be effective [42,43]. Victoria's 80 MCH Centres (across 39 regions) are a cornerstone of service provision with 96% of all first-time parents attending [34]. MCH nurses routinely establish first-time parents' groups through which education sessions are delivered. A recent prospective study [43] reported that 2/3rds of eligible first-time mothers joined these groups and that of these groups, 2/3rds were still meeting 18 months after the formal sessions had concluded. Drop out from such groups was estimated to be between 10 and 15%. That study also documents the important social environment that first-time mothers' groups provide throughout this early period of parenting. Given the stability of these pre-existing groups and the well-documented capacity of groups to support and reinforce the uptake of knowledge and skills we propose that these groups will provide an important vehicle by which we may deliver an intervention. In addition, our pilot work and engagement with local government areas has demonstrated that access to these groups via MCH nurses is feasible and that willingness of mothers within these groups to participate in research is high.

While this intervention utilises professional support beyond that currently existing in MCH Centres, it remains modest in terms of total cost of implementation and is designed for long-term sustainability, with skills required easily transferable to MCH nurses and other comparable health professionals.

The intervention draws on parenting support theory [44], which emphasizes children's psychological and behavioural goals, logical and natural consequences, mutual respect and encouragement techniques. Emphasis will be placed on parents' understanding of how improved parenting skills can facilitate the development of appropriate eating and activity behaviours in children. In the feeding domain, these approaches have been operationalised by Satter who promotes the 'Division of Responsibility in Feeding' [45] This approach has also been adopted in the US Start Health Feeding Guidelines for Infants and Toddlers [46]. Havery-Berino et al [47] report, in one of the only relevant studies in this age group (age 9 to 36 months), a positive impact on children's dietary intakes using similar approaches.

The intervention will use an anticipatory guidance framework, to coincide with opportunities to support parents regarding feeding, physical activity and sedentary behaviour issues for infants prior to their evolution. In addition, it will utilise the dynamics of existing first time parents' groups to support and reinforce the messages delivered in the intervention.

## Aims and Hypotheses

The aim of the study is to test the effectiveness of an early childhood obesity prevention intervention delivered to first-time parents and focussed on parenting skills which support the development of positive diet and physical activity behaviours, and reduced sedentary behaviours in infants from 3 to 18 months of age.

### Study hypotheses

In comparison to the control group infants, over the course of the intervention, the intervention group infants will:-

• Demonstrate greater increases in consumption of fruits and vegetables, and smaller increases in consumption of cordials, soft-drinks and juices and energy-dense snack foods.

• Demonstrate greater increases in time spent being physically active and smaller increases in time spent in sedentary behaviours, specifically TV viewing.

• Exhibit reduced incremental BMI gain.

In comparison to the control group parents, the intervention group parents will demonstrate greater increases in:-

• the frequency with which they offer fruit and vegetables, water and milk (rather than cordials, soft-drinks and juices); and smaller increases in the frequency with which they offer energy-dense snack foods to their child;

• knowledge regarding infant eating, physical activity and sedentary behaviours and greater development of positive attitudes/beliefs regarding their capacity to influence these behaviours.

• the adoption of desired feeding strategies, including the division of responsibility in feeding and in providing opportunities for modelling of healthy eating.

• the adoption of strategies, including modelling, for increasing opportunities for physical activity and reducing opportunities for sedentary behaviours.

## Design and Methods

### Overall study design

**Figure 1:**
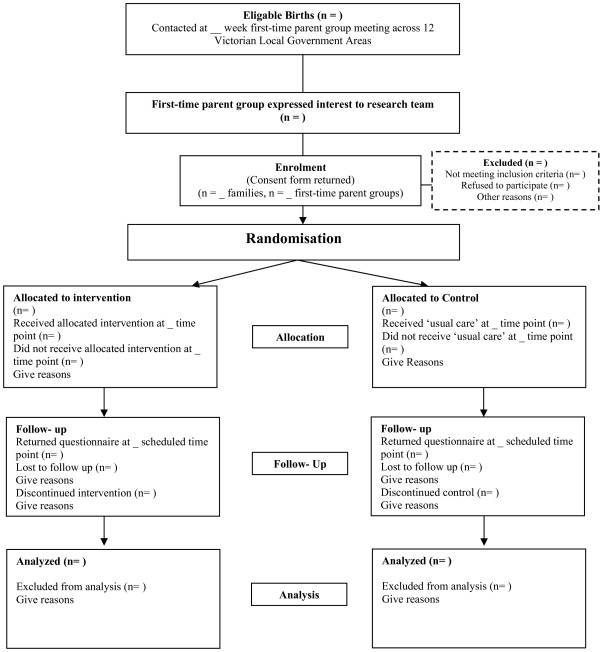
Study design.

The INFANT study is a cluster- randomised controlled trial, with first-time parent groups within local government areas, as the unit of randomization (see Figure 1). The intervention will run from three to 18 months of age (currently funded; National Health and Medical Research Council Grant No. 425801) and additional funding will be sought for follow-up to assess sustainability of outcomes. Ethical approval to conduct the study has been granted by the Deakin University Ethics Committee (ID number:EC 175-2007) and by the Victorian Office for Children (Ref : CDF/07/1138).

### Participants and recruitment

A two-stage random sampling process will be used to select first-time parent groups. At the first stage, twelve local government areas within a 60 km radius of the research centre (Deakin University in Burwood, Victoria, Australia) will be randomly selected. Local government areas within this geographical area that have an annual birth rate lower than 600 will be excluded. At the second stage, first-time parent groups within selected local government areas will be randomly selected, proportional to the total number of first-time parent groups within each area. The first-time parents group currently underway will then be invited to participate. These first-time parent groups will be accessed initially via the MCH nurse who will set up an appropriate time to invite a member of the research team to speak with the group about the study. This team member will explain the study to the group and distribute research information packages. The packages will contain general study information, consent forms and contact details of the research team so any parent is free to contact the team to discuss any concerns or unanswered questions. A majority of group members will be required to consent to allow the group to participate in the study. Non-consenting parents within participating groups will be permitted to attend the intervention sessions, but will not be required to provide data or be contacted by the research team in any other way.

### Inclusion criteria

Parents will be eligible to participate if they are able to freely give informed consent, are first-time parents, members of a participating 'first-time parents group' and are able to communicate in English.

### Exclusion criteria

Parents will be excluded from the study if they are unable to give informed consent or are unable to communicate in English. Infants with chronic health problems that are likely to influence height, weight, levels of physical activity or eating habits will be excluded from analyses but will be permitted to participate in the study.

### Sample size

A sample of 600 first time parents participating in first-time parents' groups (300 in each arm) will be recruited for the study. The sample size calculation is based on detecting changes in one of the main outcomes: dietary intake. The authors are not aware of any data providing means and standard deviations on physical activity, sedentary behaviours or body mass index in this age group. Consequently, sample size calculations were undertaken for dietary outcomes using three day weighed food data on 18 month old Australian children [13,48].

As there are no quantitative dietary recommendations for children less than four years old in Australia, we suggest a 25% increase in vegetable consumption as a minimum target on which the sample size calculations are based. Australian data show that 18-month old children eat around 32 grams of vegetables (not including potato) per day with a standard deviation of 15 g [48]. To detect an increase in vegetable consumption as small as 25% the total number of subjects required is 112 (56 in each arm). To account for within-group clustering (resulting from randomisation at the first- time parent group level) the sample size was increased by the design effect/inflation factor of 2.8, based on assumptions of each cluster consisting of approximately ten people and a conservative inter-class cluster coefficient of 0.2. The sample size estimation was further adjusted to account for attrition (40%) with the potential of loss of entire groups to follow-up and we plan to over sample by 15%. Thus the final sample is 600 (300 in each arm).

### Randomization

First-time parents groups will be randomised after recruitment in order to ensure baseline equivalence and minimise selection bias. Within each local government area, first-time parents groups will be randomly allocated to the intervention or control group in order of recruitment, using a computer generated random number schedule developed by a statistician who has no contact with the centres. Prior to the first intervention session families will be informed by letter regarding the outcome of randomisation.

### Intervention group

The intervention will be delivered by a dietitian and is comprised of six sessions delivered at three month intervals during the regular meeting time of the first-time parents' group (see Table 1). Based on an anticipatory guidance framework the intervention will incorporate a range of modes of delivery and educational strategies including brief didactic sessions, use of group discussion and peer support, exploration of perceived barriers, use of visual and written messages, follow-up delivery of messages by text-messaging and mail-outs. All educational concepts will be developed iteratively, that is, messages will be repeated and expanded upon over the course of the intervention.

**Table 1:** Intervention time-frame and focus

**Infant Age**	**Emerging Behaviours**	**Anticipatory Guidance Intervention focus**
3 mo	Early weaning and introduction of solids. Introduction of nutrient poor foods. Increased muscle control, strength and coordination.	To introduce basic concepts regarding parental feeding styles and how these might relate to beliefs about parenting. To support parents to delay weaning/introduction of solids to 6 months
6 mo	Adoption by parents of a feeding style and TV viewing habits Food rejection by infants Infant starts to: sit briefly unsupported; reaches with one hand; rolls over	To develop parents understanding regarding: *feeding styles and impact on children's eating *basic nutrition principals *sedentary behaviours in families and limits to acceptability
9 mo	Increasing use of TV Parents' increased awareness of child mobility. Infant crawls and pulls self upright and walks with handhold	To develop understanding regarding: * parental modeling of eating, sedentary and physical activity behaviours * impact of eating, activity and sedentary behaviours on health of children and adults and the provision of opportunities
12 mo & 15 mo & 18 mo	Increasing autonomy of child in eating and activity Infant stands without support and beginning to walk	Continued development of themes/skills regarding: * eating and moving for health parents and children * how to feed/how to manage food rejection and demands * providing fail-safe food and activity environments

### Control group

The control group families will receive usual care from their MCH nurse. In addition, these families will be sent general health newsletters (e.g. dental health, sun protective behaviours, general safety), and will receive Birthday and Christmas cards. These families' participation will be rewarded with gifts (to a maximum value of $15.00) on receipt of completed questionnaires.

### Measures

Parent and infant data will be collected using parent self-completion questionnaires, apart from infant dietary intake data which will be collected by telephone interview. As outlined in Table 2, all data (appropriate to the age of the child) will be collected every three months, corresponding with the six intervention sessions. Repeated data collection is necessary given the rapid changes in height, weight, eating and activity behaviours in infants. The measures collected are detailed below.

**Table 2:** Measures and time-frame for the study

Intervention timeline – year		1		2			3
Measures		Infant Age					
		3 months	6 months	9 months	12 months	15 months	18 months

Dietary Intake	Parent	✓					✓
	Child	✓	✓	✓			✓
Sedentary behaviour	Parent	✓					✓
	Child			✓			✓
Physical activity	Parent	✓					✓
	Child		✓	✓	✓	✓	✓
Family Food Environment	Parent	✓		✓			✓
Family physical activity & Sedentary Environment	Parent	✓		✓			✓
Demographic Data	Parent	✓					
Anthropometric Data	Parent	✓					✓
	Child	✓	✓	✓	✓	✓	✓

#### Dietary intake

Dietary intake for mother and father will be assessed using The Cancer Council's Dietary Questionnaire for Epidemiological Studies (Version 3) at baseline and study conclusion. This questionnaire is an updated version of the semi-quantitative food frequency questionnaire specifically developed for the Melbourne Collaborative Cohort Study [49]. Child's dietary intake will be assessed by telephone-administered multi-pass 24-hour recall with parents [50]. Visual aids will be provided to primary carers in advance of interviews to help in the estimation of quantities of food consumed. Three days of dietary data will be collected (including one weekend day) when the infant is 9 and 18 months of age.

#### Physical activity

Parents will report their frequency and duration in physical activity during the previous week using the Active Australia Survey [51]. In addition, at 12 and 18 months parents will be asked to report the number of hours their child typically spends playing outdoors on weekdays and weekend days.

#### Sedentary behaviors

Parents will be asked the amount of time the infant spends watching television on a typical weekday and on a typical weekend day [52], and to estimate the amount of time each day that the child spends immobile. Parents will also report the total time they spend watching television during their leisure-time in a typical week [53].

#### Home food environment

Three aspects of the home food environment will be assessed. Aspects of nutrition knowledge focused around nutrition targets of the intervention will be assessed modified subscales of the validated Nutrition Knowledge Questionnaire [54], Parent Feeding Style will be assess using the Child Feeding Questionnaire (CFQ) [55], the Caregivers Feeding Style Questionnaire CFSQ [56].

#### Family physical activity and sedentary environment

Parents will be asked general questions relating to their knowledge about physical activity in early childhood, their interactions with their child around physical activity and an audit checklist on the physical activity and sedentary home environment.

Standard demographic and socio-economic information will be collected by parental report. Anthropometric measures (height/length and weight) on the infant and parents will be collected by trained staff.

### Data analysis

All analyses will be conducted using the intention to treat principle. Generalized Estimating Equations (GEE) [57] will be used to fit regression models to describe the effects of the intervention on key outcome variables among parents and infants. Separate models will be fitted, to determine differences in key outcome variables in the intervention and control groups.

## Discussion

The prevalence of obesity in early childhood is rapidly increasing and is determined, in part, by eating, physical activity and sedentary behaviours. These behaviors are predominantly learnt at home in the first five years of life, and impact upon health throughout life. Given this, the early years hold promise as a time when obesity prevention may be most effective. However, research in this early period of life is lacking. This cluster-randomized trial will be one of the first to investigate whether a health promotion program delivered to first-time parents in their usual social settings can promote healthy eating, physical activity and reduced sedentary behaviours in infants. If effective, this program could protect children from the development of obesity and its associated social and economic costs. Further this study has the capacity to substantially strengthen our understanding of strategies that will promote health among families and may result in policy change both nationally and internationally.

## Competing interests

The author(s) declare that they have no competing interests.

## Authors' contributions

KC took the lead in writing and designing the study subsequently funded by a National Health and Medical Research Council Grant. She has also assisted with the modification of this grant for publication.

KH contributed to the overall concept and design of the study and assisted with the writing of the grant and its modification for publication.

DC, KB, JS, ZM provided expert input and support overall for the writing of this grant with particular emphasis on design, measures of physical activity and statistical analyses.

All authors read and approved the final manuscript.

## Pre-publication history

The pre-publication history for this paper can be accessed here:


